# Bacterial cellulose nanocrystal as drug delivery system for overcoming the biological barrier of cyano-phycocyanin: a biomedical application of microbial product

**DOI:** 10.1080/21655979.2023.2252226

**Published:** 2023-08-30

**Authors:** Heli Siti Halimatul Munawaroh, Budiman Anwar, Galuh Yuliani, Intan Cahaya Murni, Ni Putu Yunika Arindita, Gusnine Sari Maulidah, Larasati Martha, Nur Akmalia Hidayati, Kit Wayne Chew, Pau-Loke Show

**Affiliations:** a Study Program of Chemistry, Faculty of Mathematics and Science Education, Universitas Pendidikan Indonesia, Bandung, Indonesia; b Laboratory of Biopharmaceutics, Department of Pharmacology, Faculty of Pharmacy, Takasaki University of Health and Welfare, Takasaki, Japan; c Research Center for Environmental and Clean Technology, The National Research and Innovation Agency (BRIN), Tanggerang Selatan, Indonesia; d School of Chemistry, Chemical Engineering and Biotechnology, Nanyang Technological University, Singapore, Singapore; e Department of Chemical Engineering, Khalifa University, Abu Dhabi, United Arab Emirates

**Keywords:** Adsorption, bacterial cellulose nanocrystal, drug delivery system, phycocyanin, nanocellulose

## Abstract

Phycocyanin, produced by *Spirulina platensis,* has been reported as an anti-inflammatory, anti-hyperalgesia, antioxidant, anti-tumor, and anti-cancer agent. However, the ingestion of phycocyanin in the body is often hindered by its instability against gastric pH conditions. The nano-drug delivery system has developed as a promising platform for efficient drug delivery and improvement as well as drug efficacy. Bacterial cellulose nanocrystal (BCNC) has it superiority as DDS due to its inherent properties such as nanoscale dimension, large surface area, - biocompatibility, and non-toxic. To improve its mechanical properties, BCNC was crosslinked with glutaraldehyde and was analyzed as a potential candidate for DDS. The Fourier transform infrared analysis of the BCNC suggested that hydrolysis did not alter the chemical composition. The index of crystallinity of the BCNC was 18.31% higher than that of the original BC, suggesting that crystalline BC has been successfully isolated. The BCNC particle also showed a needle-like morphology which is 25 ± 10 nm in diameter and a mean length of 626 ± 172 nm. Crosslinked BCNC also had larger pores than the original BCNC along with higher thermal stability. Optimum phycocyanin adsorption on crosslinked BCNC reached 65.3% in 3 h. The release study shows that the crosslinked BCNC can protect the phycocyanin retardation by gastric fluid until phycocyanin reaches the targeted sites. This study provides an alternative potential DDS derived from natural bioresources with less expenses and better properties to promote the application of BCNC as functional nanomaterials in biomedical science.

## 1. Introduction

Cyano-phycocyanin (C-PC), a water-soluble blue pigment, is a group of phycobiliprotein which is abundantly available in *Arthrospira platensis* (*A. platensis)*. The protein is attached to the pigment and consists of glutamic acid, alanine, leucine, aspartic acid, isoleucine, serine, arginine, glycine, and threonine. This pigment–protein complex has been reported in works contributing to the pharmaceutical effects of *A. platensis* [1]. Recent studies have shown that C-PC has an anti-inflammatory activity [2,3] and is antioxidant [4], anti-tumorigenic [5], and anti-cancer [5,6]. However, when C-PC is taken as a supplement or drug, its activity is often hindered by its susceptibility to gastric fluid due to its instability at conditions below pH of 4 [7]. Therefore, the use of a drug delivery system (DDS) for delivering drugs into specific targeted tissue represents an option to overcome this problem [8,9].

DDS designates an advanced method for targeted or specific delivery of a pharmaceutical substance throughout or into the body to maintain its efficacy and prevent its retardation before reaching the desired targeted sites. Drug carrier performs its action by regulating the rate, time, dosage, duration, and location of drug release in the body. This system can also overcome solubility problems, and can protect the drug from external environment such as changes in pH while reducing the dosage disposal by controlling the drug release [10,11]. Among numerous routes, oral intake is one of the most attractive routes for drug administration into the gastrointestinal (GI) tract. Oral route can be used for both systemic drug delivery and local gastrointestinal treatment. This route is most preferred for drug carriage due to its noninvasive properties, cost-effectiveness, relatively ease-of-use, and convenience for self-consumption. However, the GI tract physiological milieu can also affect the stability and solubility of the drugs [12–14].

Cellulose has several applications in biomedicine and pharmacology due to its distinctive properties, such as high water retention capacity, high wet strength, low density, biocompatibility, non-toxicity, and biodegradability [15]. The improvement of technology has led to the finding of nanometer-sized cellulose or nanocellulose which provided new generation of cellulose for more advanced application. In pharmaceutical industries, nanocellulose and its derivatives have been widely employed as pharmaceutical carrier (matrices) or excipients to condense the loaded drugs [16,17]. Nanocellulose can be obtained from native cellulose through various methods of extraction [18]. The crystallinity of cellulose can also be increased by hydrolysis, generally by acid hydrolysis, which results in cellulose nanocrystals (CNCs) [19]. Recently, researchers have been starting to focus on natural biopolymers that are sustainable, low cost, and environmental friendly [20]. Thus, bacterial cellulose (BC) began to arise as a replacement for plant cellulose. Bacteria produces BC from simple sugar to build up the nanofiber. Hence, the resulted cellulose product will be pure and free from contaminants. On the contrast, plant cellulose always contains hemicellulose and lignin and requires additional chemicals for purification which can pollute the environment. Pineapple plantations in Indonesia cover an area of more than 28,000 hectares, where the total production of pineapple is about 20 tons per hectare per year with the percentage of pineapple peel waste is around 23% [21]. The accumulating waste of pineapple peel can be used as a potential source of glucose since a huge amount of these wastes is regularly disposed of, and by transforming it into useful materials, the concept of sustainable development can also be achieved [22]. Pineapple peel waste contains sugar which is useful as a medium for BC biosynthesis by *Gluconacetobacter xylinus* [21].

The use of bacterial cellulose nanocrystal (BCNC) as a DDS agent has several advantages, such as good biocompatibility, non-toxicity, biodegradability [23,24], and negatively charged with high surface area. These advantages enable more effective cellulose nanocrystal (CNC) binding to the drug molecule to improve the loading efficiency [25]. To improve its mechanical properties, BCNC must be crosslinked with a crosslinker [26]. Glutaraldehyde (GTA) is one of the most widely used crosslinking agents, due to its high efficiency [27,28]. In addition, GTA has been frequently used as a crosslinking agent than any other crosslinker since it is low-priced, highly soluble in aqueous solution, and readily available [29]. Crosslinking with GTA is clinically accepted and has many benefits despite several reports of its cytotoxicity [29,30].

This present work focuses to develop the BCNC with cheaper and better features by crosslinking with GTA. The GTA will form links in an intermolecular and intramolecular through the formation of covalent bonds that mainly involve the amino groups of the polysaccharide. The prospect and effective application of BCNC as DDS was evaluated using C-PC extract. The BCNC was used in an attempt to increase and control the extract of C-PC delivery to intestinal tract. The C-PC was loaded to the BCNC to develop a carrier which is capable of preserving the C-PC stability in the GI tract and increase the drug release to improve its efficacy. The BCNC crosslinked with GTA was also developed to prevent the water-soluble C-PC protein to degrade by the strong acid and digestive enzyme normally present in the gastric environment and to improve its absorption in GI tract. The modification of GTA-BCNC provides an alternative potential local as well as temporal controlled release of the drug that has never been reported in the previous studies. This current work also promotes specific features for the production of BCNC with cheaper and better properties and the applications of BCNC in the field of functional nanomaterials.

## 2. Materials and methods

### 2.1. Materials

Chemicals of technical grade and no prior treatment used for biosynthesis of BC and isolation of nanocrystal cellulose (NCC) were procured from local supplier, while the chemicals with pro analysis grade used for analysis were purchased from Sigma and Aldrich chemical company. *Gluconacetobacter xylinus* bacteria was obtained from local suppliers. Waste pineapple peels was obtained from the pineapple plantation in Subang, West Java Indonesia. *Spirulina platensis* biomass were obtained from Biology Department of Padjadjaran University. The cellulose membrane was procured from the Chemistry Research Laboratory, Bandung, Indonesia.

### 2.2. Preparation of BCNC crosslinked with GTA

Production of BC was performed according to a previous study [21]. Isolation of BCNC includes the hydrolysis using 50% sulfuric acid with the acid–BC ratio of 50 ml/g and proceeded at 45°C under constant stirring for 40 min. The optimum conditions were taken from the optimized previous study [21]. The hydrolysis process was stopped by quenching the samples into a tenfold volume of deionized water. The suspension of cellulose was centrifuged at 4000 × g for 10 min to get the sediment which was then dialyzed using CelluSep T4 Regenerated Cellulose Tubular Membrane MWCO 12.000–14.000 against deionized water until the dispersion reached pH ~ 6. Finally, the suspension was sonicated for 10 min using a Branson 2510 Ultrasonic Cleaner. The yields of suspension were then freeze-dried (OPERON Freeze Dryer Bench Top FDB-5003) to obtain BCNC powder.

Synthesis of crosslinked BCNC with GTA was performed using a previous method [31]. The BCNC suspension was placed into a beaker and GTA solution was added as a crosslinker, with several variations in the mass fractions of 1 m/m% and 2 m/m%, which was stirred for 1 h. Then, the suspension was freeze-dried using OPERON Freeze Dryer Bench Top FDB-5003.

### 2.3. Characterization of BCNC

#### 2.3.1. Scanning Electron Microscopy (SEM) and Transmission Electron Microscopy (TEM)

The SEM and TEM analyses conditions were performed with reference to a previous method [32]. The morphology of BC was observed by SEM examination. Dry samples were coated using an ion sputter with gold or palladium. The prepared samples were imaged using a JEOL JSM-6510 SEM set to 8–10 kV. The morphology and particle size distribution of BCNC were determined using a JEOL JEM-1400 TEM by depositing the suspension of BCNC onto carbon-coated microscope grids and allowed to dry. By measuring at least 175 distinct particles, the size of the particle was calculated statistically from the SEM or TEM analysis findings.

#### 2.3.2. Fourier Transform Infrared (FTIR)

FTIR spectroscopy was determined by referring to a previous method [32]. The spectra of BC and BCNC were measured using infrared spectrometer and subsequently recorded at room temperature. The spectra were generated from a Prestige Shimadzu FTIR spectrometer instrument. To find information on changes in the chemical composition, the samples were formed into KBr pellets and evaluated over the region of 4500–450 cm^−1^ at a scanning resolution of 2 cm^−1^. The ratio of crystallinity of BCNC was also evaluated by recording the peaks at 1372 cm^−1^ (A1372) and 2900 cm^−1^ (A2900) and calculated by using equation (1) [33]:

(1) $$CrR=\frac{A_{1372}}{A_{2900}}$$

where *CrR* represents the crystallinity ratio while A_1372_ and A_2900_ are the peaks at 1372 cm^−1^ and 2900 cm^−1^, respectively.

#### 2.3.3. X-ray Diffraction (XRD)

To demonstrate the crystallinity of cellulose, XRD measurement was utilized. XRD examination was performed on BCNC using a Philips PW 1835 diffractometer with CuK radiation (=1.54) in the two ranges of 5–90° to investigate its crystallinity. The measurement was conducted at a voltage and current of 40 kV and 30 mA, respectively. The index of crystallinity (CrI) was calculated using equation (2) [34]:

(2) $$CrI=[1-(I_{am}/I_{200})]x100\%$$

I_200_ is the maximum intensity of the crystal lattice diffraction peak (around 2θ = 22.5° for cellulose I) and I_am_ is the diffraction intensity of the amorphous part (around 2θ = 18° for cellulose I).

#### 2.3.4. Thermogravimetric Analysis (TGA)

TGA was performed using TG/TGA Shimadzu DTG 60A to investigate the thermal stability of BC, BCNC, and BCNC-GTA. Each sample was heated from 27°C to 550°C at a rate of 5°C min^−1^ under a nitrogen environment.

#### 2.3.5. Swelling rate

The degree of swelling was measured using a previous method [35]. Each sample (0.01 g) was poured into 100 ml of distilled water. Data was recorded at a certain time interval and the water uptake of sample was calculated using equation (3):

(3) $$Q\left(g\right)=\left(m_{2}--m_{1}\right)/m_{1}$$

where Q represents the swelling rate or water absorbency while m_1_ and m_2_ are the dry weight of the sample before and after swelling, respectively. Swelling rate measurements of the BCNC were conducted three times.

### 2.4. Water absorbency

Measurement of water absorbency was performed using previous method [35]. The sample (0.01 g) was immersed in 100 ml of distilled water under room temperature for 4 h to achieve swelling equilibrium, then separated from the medium by sifting through a 200-mesh nylon sieve. Water absorption (Q) was calculated using equation (3). Water absorbency measurements of the BCNC were conducted in triplicates.

### 2.5. Extraction and purification of Cyano-Phycocyanin (C-PC)

C-PC extraction and purification were performed according to a previous study [36–38]. The C-PC was purified using ammonium sulfate ((NH_4_)_2_SO_4_) precipitation by referring to previous experiments [36]. The purification process was carried out in triplicates by subjecting the C-PC to 25% ammonium sulfate and 50% ammonium sulfate. A greenish residue and blue supernatant were obtained at 25% saturation. The yields of blue supernatant were then subjected to a 50% ammonium sulfate to obtain blue residue and colorless supernatant. The yields of residue from 50% saturation were dissolved with phosphate buffer, then continued with dialysis using cellulose membrane to remove the remaining ammonium sulfate. Purification of the extracted dialyzed C-PC extract was conducted for 6 days by replacing the pH 7 phosphate buffer solvent once per day. After the dialysis, the C-PC content was freeze-dried.

### 2.6. Adsorption of C-PC extract

C-PC extract adsorption on BCNC was measured isothermally at 25°C under pH 7.5 condition [39]. The adsorption study was performed by referring to the previous study [40]. A known CNC amount of 20 mg was poured to a series of Erlenmeyer flasks each containing a mixture of 12 mL of a solution with 62 ppm C-PC extract. All flasks were placed in a thermostatic water bath shaker for 3 h.

The concentration of C-PC in the solution before (C_o_) and after reaching equilibrium concentration (C_e_) was obtained using a Shimadzu UV Mini 1240 at range of wavelength of 200–800 nm. The amount of C-PC extract absorbed by CNC at equilibrium condition (q_e_) was calculated using equation (4):

(4) $$q_{e}=\frac{\left(C_{o}-C_{e}\right)}{m}V$$

Units of m and V represent the mass of the CNC and the volume of the solution. The adsorption study was carried out in three independent experiments.

Adsorption isotherms are usually used to describe the interactions that occur between the adsorbent and the adsorbate. In this study, Langmuir and Freundlich isotherms were used; thereafter, a more suitable isotherm was chosen. Langmuir’s equation is presented in equation (5):

(5) $$q_{e}=q_{max}\frac{K_{L}C_{e}}{\left(1+K_{L}C_{e}\right)}$$

The q_max_ represents the adsorption capacity, while the K_L_ characterizes adsorption affinity of the adsorbent [41,42].

The Freundlich equation represents the adsorption of the liquid phase on heterogeneous surfaces. The formula of the Freundlich’s equation is presented in equation (6):

(6) $$q_{e}=K_{F}{C_{e}}^{1/n}$$

where K_F_ signifies the adsorption capacity and n denotes the heterogeneity of the system with a value ranging from 1 to 10. The higher the value of n, the more heterogeneous a system is. Freundlich’s isotherm model is used to predict a multilayer adsorption [43].

### 2.7. C-PC extract release studies

The drug release studies were measured at 37°C in phosphate buffer (PB) medium with pH values of 7.2 and 2.1. A sample of BCNC loaded with C-PC extract (0.02 g) was subjected to a series of beaker glass containing 15 mL of phosphate buffer solution. All flasks were put into a water bath (Memmert WB-14 thermostatic shaker) at 37°C. During the drug release process, the beaker glass was shaken at 170 RPM. Each beaker glass was removed from the system one by one at every certain interval. The concentration of C-PC extract release was recorded as a function of time (C_t_) using Shimadzu UV Mini 1240 at a wavelength of 200–800 nm. The blank of phosphate buffer solution was used as a control solution for UV analysis. Drug release studies were carried out in triplicates.

## 3. Results and discussion

The characteristics of the isolated CNC from BC through the acid hydrolysis method to develop a potential drug carrier (DDS) from cellulose-based material were performed in this study. The developed DDS of BCNC was characterized to determine its features for establishing the method for the production of BCNC as well as to evaluate its potential as a drug carrier for phycocyanin.

### 3.1. Characteristics of BCNC

**Figure 1.**
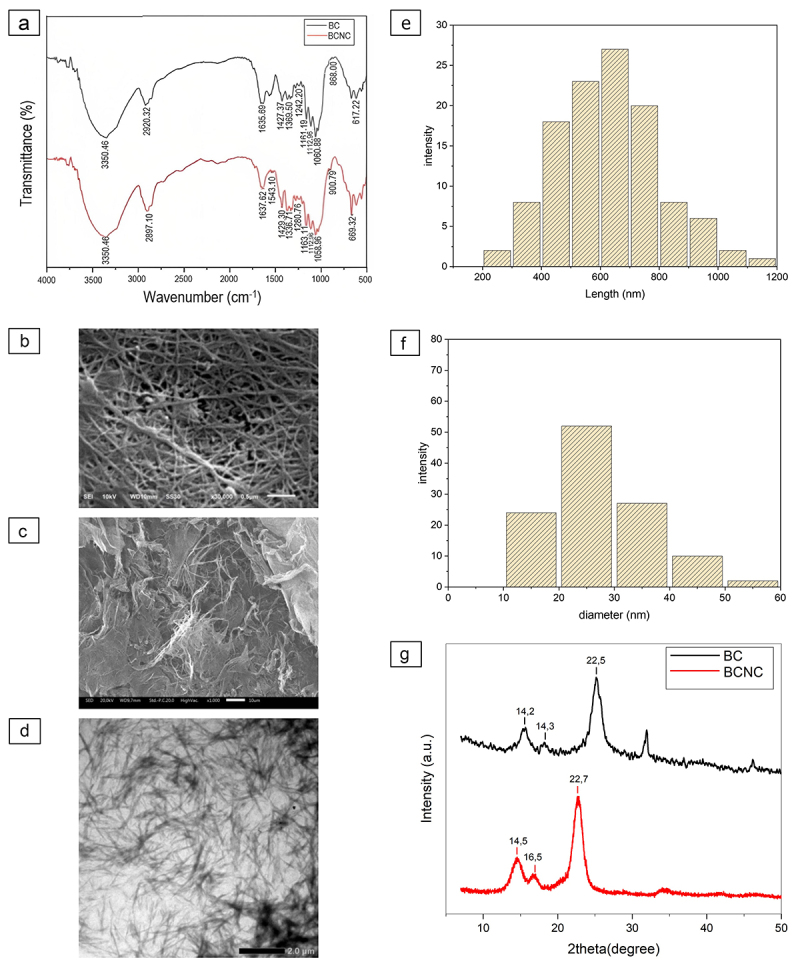
Characteristics of bacteria cellulose and the synthesized BCNCs. (a) The infrared spectra of BC and BCNC; (b) SEM image of BC; (c) SEM image of BCNC; (d) TEM image of BCNC; (e) particle size distribution of BCNC (length); (f) particle size distribution of BCNC (diameter); and (g) XRD of BC and BCNC.

The comparative analysis of the BC and BCNC FTIR spectra identified the appearance of peak near 3440–3400 cm^−1^, which resembles the –OH groups. The peak around 1640 cm^−1^ in the FTIR spectra of BC and BCNC corresponds to the absorption of water. Both the peak absorbances are ascribed to the stretching of hydrogen bonds and bending of hydroxyl (OH) groups bound to the cellulose structure [32,44]. The FTIR spectrum in Figure 1a shows that the spectrum of BCNC is similar to that of BC, suggesting that the hydrolysis of BCNC using acid did not significantly affect the chemical composition. There was a slight shift found at the peaks around 2900 cm^−1^ and 1430 cm^−1^, which indicates that the two substances have different crystallinities [32]. Using equation (1), it was obtained that the ratio of crystallinity (CrR) of BC and BCNC are 1.0239 and 1.0492, respectively, indicating that BCNC has higher crystallinity compared to the original BC.

**Figure 2.**
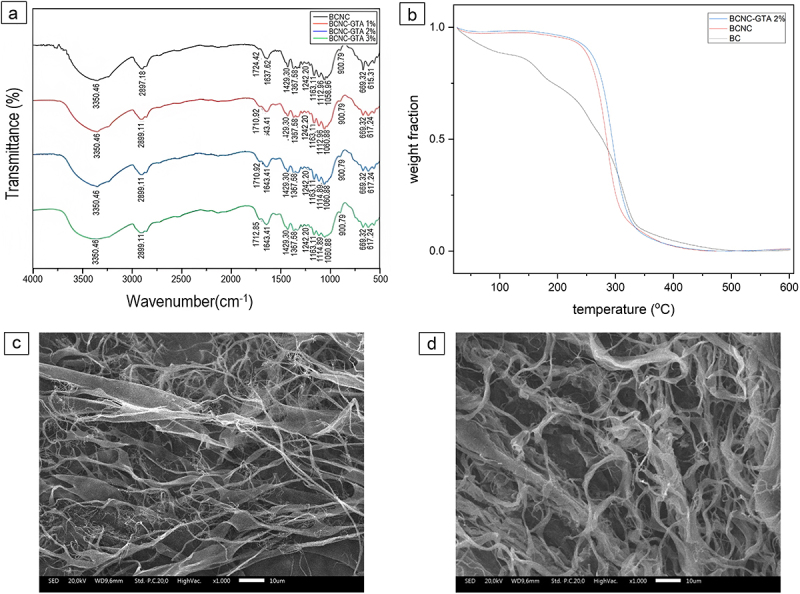
Characteristics of the synthesized BCNCs crosslinked with GTA at different weight percent. (a) FTIR spectra of BCNC, BCNC-GTA 1%, BCNC-GTA 2%, and BCNC-GTA 3%; (b) TGA curve of BC, BCNC, and BCNC-GTA 2%; (c) SEM image of BCNC-GTA 1%; and (d) SEM image of BCNC-GTA 2%.

Some absorption peaks under the wavelength range of 1700–850 cm^−1^ in the FTIR spectra of BC and BCNC demonstrated the polymorph structure of the cellulose. The absorbance at about 1420 and 1155 cm^−1^ in the spectra of BC and BCNC suggested the existence of the cellulose I [33]. According to Carrillo et al. [45], if the content of cellulose I had been dominant, the peaks at 1420 and 1155 cm^−1^ would have been shifted at 1430 and 1162 cm^−1^, respectively. In this work, the absorption peaks are detected at 1427 and 1161 cm^−1^ for the original BC and 1429 and 1163 cm^−1^ for BCNC. The detection of peak at 1280, 1317, and 1336 cm^−1^ in both original spectra of BC and BCNC also supported the major occurrence of cellulose I. The polymorph structure of BC and BCNC were also observed in the XRD diffractograms. GTA was used as a crosslinking agent for chemical crosslinking of cellulose chains since it is less expensive, readily available, highly soluble [29], highly efficient [27,28], and clinically accepted [29,30]. The FTIR spectra of BCNC-GTA are shown in Figure 2a. The BCNC-GTA shows two significant differences in the FTIR spectra, compared to that of BCNC. The first one is a decrease of the O–H band intensity at 3350 cm^−1^ as the GTA concentration increases, and the other is the presence of the aldehyde peak (CHO) at 1710–1712 cm^−1^ [46]. These differences indicated that the cellulose chains have been successfully crosslinked with GTA. Moreover, the spectrum of BCNC-GTA has a higher peak intensity at 1060–1163 cm^−1^, which is ascribed to C–O stretching. This also proves that GTA is linked to cellulose chains and the double bonds (C=O) are open. The linkages occurred between the reactive C-6 hydroxyl groups of cellulose and the hydrated regions of GTA [47]. This peak overlapped with the water absorption peak of the cellulose; therefore, the overall intensity of these peaks was increased after crosslinking [32,48].

Figure 1d shows the needle-like shape morphology of BCNC using TEM analysis. Needle-like morphology is a typical morphology for CNC [49]. The length and average diameter of BCNC particles obtained from TEM images were 626 ± 172 and 25 ± 10 nm as shown in Figure 1(e,f). These results suggested that acid hydrolysis with a concentration of 50% was necessary for optimum isolation of BCNCs from BC. Upon hydrolysis, the BC was converted to BCNC which resulted in typical needlelike morphology. This is expected for crystalline BC due to higher crystalline region that can survive acid attacks better than the amorphous region. Higher degree of amorphous region and prolonged hydrolysis rate would result in a spherical morphology. BC has higher purity than plant cellulose, and delignification process is not required in the processing that leads to conservation of the crystalline structure, resulting in the typical rod/needlelike morphology upon the acid hydrolysis.

As shown in Figure 1b, the BC film has morphology of a ribbon-like microfibrils with length about several micrometers and an average diameter of 51 ± 25 nm. Figure 1d shows the TEM image of BCNC where it has needle-like structure morphology. This structure is a typical morphology for CNC^40^. As shown in Figure 1(e,f), the length and average diameter of BCNC particles obtained from TEM analysis were 626 ± 172 and 25 ± 10 nm, respectively. Figures 1c and 2(c,d) show the freeze-dried BCNC, BCNC-GTA 2%, and BCNC-GTA 3%, respectively. All of these showed a porous structure; however, there was a gradual increase in the pore size which allows more water to enter the pores during the swelling process.

**Figure 3.**
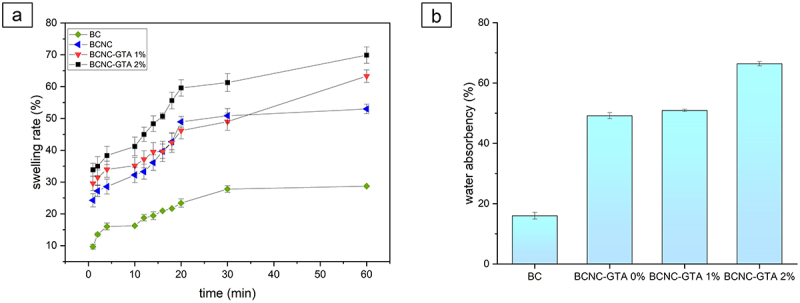
The properties of the varying BCs and enhanced BCNCs crosslinked with GTA. (a) Swelling test and (b) water absorbency.

On the other hand, the addition of GTA as a crosslinker made the band wider which could have an impact on the mechanical properties of BCNC. The mechanical properties of BCNC-GTA are predicted to be higher due to the width of these band, although the pore size also increase. This is shown in the results of swelling ratio (Figure 3a) where the rate of expansion and water absorption (Figure 3b) in BC were very low, whereas the highest rate of 2% was shown in BCNC-GTA. Swelling and water absorption rate were increased to 81.87% and 66.40%, respectively, mainly due to the fact that the surface of BCNC contains plenty of hydrophilic groups (–OH) which causes high hydrophilicity. Consequently, the effect of crosslinking GTA caused an improvement of the mechanical properties of BCNC-GTA into the highest rate of 2%; therefore, the swelling of BCNC was preferred to be conducted in the water. Crosslinking between nanocellulose and GTA provides more intermolecular associations, thus affect the pore structure and size, which then affect the application performance as a DDS.

Structurally, cellulose fibers contain amorphous and crystalline parts. The amorphous part has an irregular structure, whereas the crystalline part has a regular structure. During acid hydrolysis, the amorphous part of the cellulose fiber will be hydrolyzed into glucose, while the crystalline part remains intact due to its excellent stability under acidic condition [49,50]. The XRD patterns of BC and BCNC are shown in Figure 1g, which showed four characteristic peaks at the diffraction peak around 2θ = 14.5°, 17°, and 22.5°. These peaks were in the same position as the cellulose I crystal peaks at around 2θ = 15.9° 16.4°, 22.6°, and 34.6°. Additionally, the diffractogram of XRD of BC and BCNC at around 2θ = 22.6° showed sharper peaks for BCNC than that of BC. The higher degree of crystallinity of BCNC compared to BC was indicated by the sharper diffraction peak of BCNC than BC [49]. The crystallinity indexes of BC and BCNC were 69.49% and 87.8%, respectively. The index of crystallinity of the BCNC was 1.26 times higher than that of BC due to the removal of amorphous fraction by sulfuric acid hydrolysis [51].

The thermal stability of polymeric materials depends on the characteristics of the sample and the interactions of different macromolecules. Chain breaking or macromolecular bond dissociation occurs when the heat energy applied exceeds the bond dissociation energy of the individual chemical bond [52].

The weight loss of cellulose nanoparticles with increasing temperature and its degradation rate are shown in Figure 2b. The thermal stability indicates the BCNC potential utilization in various applications. Although this feature is not crucial in the drug delivery application due to lower application temperature, this may be important in the processing and storage of the material. The thermal decomposition of water of the samples was observed in the range of 50–200°C. In addition, the decomposition of cellulose into small molecular weight compounds was observed in the range of 250–350°C [49]. We observed that the main degradation temperature was shifted to the higher temperature range in 2% BCNC-GTA, which was then followed by BCNC and BC, respectively. GTA is an effective short-chain molecule and crosslinker with high crosslink density; hence, short GTA crosslinks limit the freedom of movement of nanocellulose molecules, resulting in a high transition temperature and higher thermal stability [27].

### 3.2. Characteristics of C-PC extract

**Figure 4.**
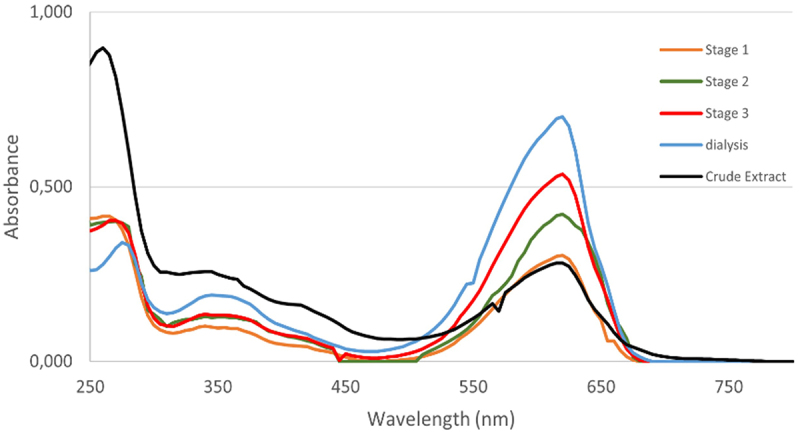
UV–vis spectrum of extraction and purification stages of C-PC compound.

To demonstrate the characteristics of C-PC extract, UV–vis spectroscopic method was utilized. The crude extract of C-PC obtained from the biomass extraction of *Spirulina platensis* has a bluish green color. The results of the UV-Vis spectroscopic analysis of the crude extract of C-PC are shown in Figure 4. The maximum absorption occurs at a wavelength of 620 nm (C-PC), and two other smaller peaks at a wavelength of 415 nm (chlorophyll) and 345 nm (billin, carotenoid), respectively.

Furthermore, the success of the purification protocol was also revealed by the UV–vis overlay spectrum which is shown in Figure 4. A sharp increase in the single peak at 620 nm indicates the maximum absorbance of C-PC, while a decrease in absorbance at 280 nm suggests the removal of proteins other than C-PC [36]. The purity of the C-PC extract preparation was evaluated based on the ratio of absorbance of phycocyanobilin at 620 nm, A_620_ and aromatic amino acids to all proteins in the sample preparation at 280 nm, A_280_. C-PC extract preparations with A_620_/A_280_ greater than 0.7 are considered suitable as food grade and A_620_/A_280_ greater than 4.0 are considered as analytical grade due to its high purity [53]. Based on the results of the purification stage of up to 4 (dialysis), C-PC extract was concluded to be successfully purified into a food grade ratio of 2.105.

### 3.3. Adsorption of C-PC extract

**Figure 5.**
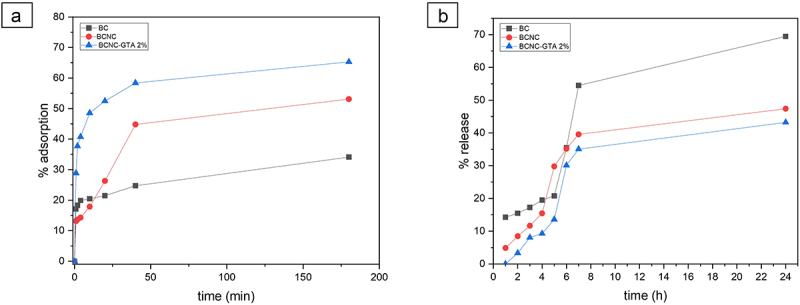
Adsorption kinetics of C-PC at different parameters. (a) The effect of contact time on the percentage of C-PC adsorption, and (b) release study of C-PC extract under pH 7.2 condition.

The effect of contact time on C-PC extract adsorption in BCNC was conducted by varying the contact time as shown in Figure 5a. The chemical structure characterization indicated that the adsorption of C-PC extract is largely a consequence of hydrogen bonding interactions, electrostatic interactions, and van der Waals forces between the C-PC and the cellulose [54–56].

The percentage of C-PC extract adsorption was increased along with the increasing contact time, while the adsorbate concentration was decreased. Additionally, the optimum value indicated that a higher adsorption efficiency was detected. C-PC extract adsorption increased along with increasing time until it reached the optimum contact time of 40 min with the percentage of 58.4%, due to the presence of highly reactive sites of hydroxyl groups on the surface of BCNC. As shown in Figure 5a, the highest adsorption occurred at BCNC-GTA of 2%, which suggested that the crosslink was successfully enhanced by the mechanical properties of BCNC. Moreover, after 40 min, the adsorption process became slow since the adsorbent has an active side which is then filled, thus slowing down the adsorption process.

**Figure 6.**
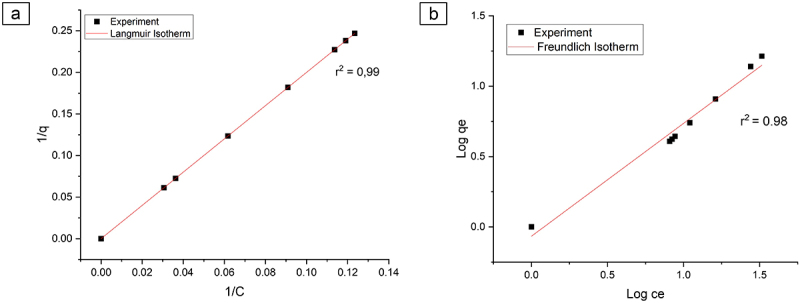
Langmuir and Freundlich isotherm adsorption.

The application of the isotherm model can be observed from the value of r^2^ (coefficient of determination) of each model. The r^2^ value in the Langmuir’s model is preferable compared to the Freundlich’s model (Figure 6) with an r^2^ value of 0.99 which confirms that the experimental data can be accurately described by the Langmuir model isotherm. We hypothesize that the adsorption mechanisms occurred with a monolayer. Another hypothesis for the Langmuir’s model is that the reaction was reversible and the interaction between the adsorbed species did not occur [41]. The adsorption on the surface was localized, suggesting that atoms or molecules were adsorbed at a certain position and did not migrate [57].

### 3.4. C-PC extract release study

In vitro release study of C-PC extract on matrix (BC, BCNC, BCNC-GTA 2%) was studied under phosphate buffer conditions at pH = 2.1 and pH = 7.2. The experimental results showed that the release of the C-PC extract from the matrix at pH 2.1 did not show the release rate in acidic media before the 7-h release study. After 24 h, the amount of C-PC extract released from the BC, BCNC, and BCNC-GTA was 40.79%, 12.91%, and 10.23%, respectively. This may be due to the stability of nanocellulose under acidic conditions. The size of the CNC aggregate increased significantly with the addition of HCl. The CNC has higher ratio of surface area to volume than BC, enabling high loading and binding capacity [58]. Additionally, nanocellulose has stability in a negative charge, but under acidic conditions, the zeta potential of nanocellulose had increased. This is due to the interferences with the crystallinity of nanocellulose, resulting in the loss of crystallinity of nanocellulose, and, therefore, it becomes less sensitive to ions [59,60].

However, the release profile of C-PC extract drastically changed when the matrix was incubated under phosphate buffer conditions (pH = 7.2) (Figure 2), due to the drug diffusion into the external medium. The release of the drug was faster for BC as there was poor swelling of the BC. As seen in Figure 3d, BCNC-GTA 2% has the best swelling ratio. After 24 h, the amount of C-PC extract released from the BC, BCNC, and BCNC-GTA was 69.46%, 47.39%, and 43.21%, respectively. The introduction of GTA reduced the hydrophilicity of the BCNC and improved the long-time stability, resulting in slower drug release [46]. These results suggest that the GTA modulated the denser structure of BCNC and led to a slow sustained release state [61]. Without the GTA, the C-PC molecules inside will be driven closely to each other, and, therefore, they readily come in contact with the dissolution medium, leading to a fast release than that of BCNC-GTA [62]. In contrast, the modified BCNC with GTA promoted stronger bond of C-PC with BCNC-GTA and sustained the drug release [62]. Those results demonstrated that the BCNC and BCNC-GTA may be suitable alternatives for drug delivery that can withstand the gastric fluid to ensure C-PC extract is released on the targeted organ.

## 4. Conclusion

The present work has explored the potential of crosslinked BCNC as a candidate for DDS. The morphology of BCNC extracted from BC has a needle-like structure, with an average diameter and length of 25 ± 10 nm and 626 ± 172 nm, respectively, and an average aspect ratio (L/D) of 25 ± 1. The acid hydrolysis using sulfuric acids produced BCNC with higher crystallinity index than the original BC. The optimum adsorption of phycocyanin reached the crosslinked BCNC at 65.3% in 3 h. The release study shows that the crosslinked BCNC as drug delivery of phycocyanin can prevent its degradation from gastric fluid that can cause the release of phycocyanin on the target. The current work establishes the development of BCNC as an alternative promising DDS originating from less-expensive materials with a cost-effective method. This study also considers the potential green production of nanomaterials that require less involvement of hazardous chemicals. Further exploration and modification of BCNC as drug carrier for insoluble bioactive compounds and toxicity assessment of the developed DDS will be needed to provide safer, cheaper, and better properties of drug carrier.

## Data Availability

Data are available on reasonable request from the authors.

## References

[1] Gorgich M, Passos MLC, Mata TM, et al. Enhancing extraction and purification of phycocyanin from Arthrospira sp. with lower energy consumption. Energy Rep. 2020;6:312. doi: 10.1016/j.egyr.2020.11.151

[2] Izadi M, Fazilati M. Extraction and purification of phycocyanin from spirulina platensis and evaluating its antioxidant and anti- inflammatory activity. Asian J Green Chem. 2018;2:364. doi: 10.22034/AJGC.2018.63597

[3] Opretzka LCF, Do Espírito-Santo RF, Nascimento OA, et al. Natural chromones as potential anti-inflammatory agents: pharmacological properties and related mechanisms. Int Immunopharmacol. 2019;72:31. doi: 10.1016/j.intimp.2019.03.044 30959369

[4] Mitra S, Siddiqui WA, Khandelwal S. C-Phycocyanin protects against acute tributyltin chloride neurotoxicity by modulating glial cell activity along with its anti-oxidant and anti-inflammatory property: a comparative efficacy evaluation with N-acetyl cysteine in adult rat brain. Chem Biol Interact. 2015;238:138. doi: 10.1016/j.cbi.2015.06.016 26079211

[5] Jiang L, Wang Y, Yin Q, et al. Phycocyanin: a potential drug for cancer treatment. J Cancer. 2017;8:3416. doi: 10.7150/jca.21058 29151925 PMC5687155

[6] Wen P, Hu TG, Wen Y, et al. Targeted delivery of phycocyanin for the prevention of colon cancer using electrospun fibers. Food Funct. 2019;10(4):1816. doi: 10.1039/C8FO02447B 30806395

[7] Minic SL, Stanic-Vucinic D, Mihailovic J, et al. Digestion by pepsin releases biologically active chromopeptides from C-phycocyanin, a blue-colored biliprotein of microalga Spirulina. J Proteomics. 2016;147:132. doi: 10.1016/j.jprot.2016.03.043 27084687

[8] Martinho N, Damgé C, Reis CP. Recent advances in drug delivery systems. J Biomater Nanobiotechnol. 2011;2(05):510. doi: 10.4236/jbnb.2011.225062

[9] Patra JK, Das G, Fraceto LF, et al. Nano based drug delivery systems: recent developments and future prospects. J Nanobiotechnol. 2018;16(1). doi: 10.1186/s12951-018-0392-8 PMC6145203 30231877

[10] Korting HC, Schäfer-Korting M. Carriers in the Topical Treatment of Skin Disease. Drug Delivery. Vol. 197, Handbook of Experimental Pharmacology. Springer; 2010. doi:10.1007/978-3-642-00477-3_15 20217539

[11] Gupta R, Rai B. Computer-aided design of nanoparticles for transdermal drug delivery. 2020;225–518. doi: 10.1007/978-1-4939-9798-5_12 31435925

[12] Shreya AB, Raut SY, Managuli RS, et al. Active targeting of drugs and bioactive molecules via oral administration by ligand-conjugated lipidic nanocarriers: recent advances. AAPS Pharm Sci Tech. 2019;20(1). doi: 10.1208/s12249-018-1262-2 30564942

[13] Homayun B, Lin X, Choi HJ. Challenges and Recent progress in oral drug delivery systems for biopharmaceuticals. Pharmaceutics. 2019;11(3):129. doi: 10.3390/pharmaceutics11030129 30893852 PMC6471246

[14] Araújo F, Das Neves J, Martins JP, et al. Functionalized materials for multistage platforms in the oral delivery of biopharmaceuticals. Prog Mater Sci. 2017;89:306. doi: 10.1016/j.pmatsci.2017.05.001

[15] Ates B, Koytepe S, Ulu A, et al. Chemistry, structures, and advanced applications of nanocomposites from biorenewable resources. Chem Rev. 2020;120(17):9304. doi: 10.1021/acs.chemrev.9b00553 32786427

[16] Bashari A, Shirvan AR, Shakeri M. Cellulose-based hydrogels for personal care products. Polym Adv Technol. 2018;29(12):2853–2867. doi: 10.1002/pat.4290

[17] Sun B, Zhang M, Shen J, et al. Applications of cellulose-based materials in sustained drug delivery systems. Curr Med Chem. 2018;26:2485. doi: 10.2174/0929867324666170705143308 28685683

[18] Gupta GK, Shukla P. Lignocellulosic biomass for the Synthesis of nanocellulose and its eco-friendly advanced applications. Front Chem. 2020;8(1). doi: 10.3389/fchem.2020.601256 PMC7793639 33425858

[19] Camacho M, Regina Y, Ureña C, et al. Synthesis and Characterization of nanocrystalline cellulose derived from pineapple peel residues. J Ren Mater. 2017;5:271. doi: 10.7569/JRM.2017.634117

[20] Noor MHM, Ngadi N, Luing WS. Synthesis of magnetic cellulose as flocculant for pre- treatment of anaerobically treated palm oil mill effluent. Chem Eng Trans. 2018;63:589. doi: 10.3303/CET1863099

[21] Anwar B, Bundjali B, Arcana IM. Isolation of cellulose nanocrystals from bacterial cellulose produced from pineapple peel waste juice as culture medium. Procedia Chem. 2015;16:279. doi: 10.1016/j.proche.2015.12.051

[22] Mansor AM, Lim JS, Ani FN. Characteristics of Cellulose, Hemicellulose and Lignin of MD2 Pineapple Biomass. Chem Eng Trans. 2018;63:127. doi: 10.3303/CET1972014

[23] Qing W, Wang Y, Wang Y, et al. The modified nanocrystalline cellulose for hydrophobic drug delivery. Appl Surf Sci. 2016;366:404. doi: 10.1016/j.apsusc.2016.01.133

[24] Zainuddin N, Ahmad I, Kargarzadeh H, et al. Hydrophobic kenaf nanocrystalline cellulose for the binding of curcumin. Carbohydr Polym. 2017;163:261. doi: 10.1016/j.carbpol.2017.01.036 28267505

[25] Thomas D, Latha MS, Thomas KK. Synthesis and in vitro evaluation of alginate-cellulose nanocrystal hybrid nanoparticles for the controlled oral delivery of rifampicin. J Drug Deliv Sci Technol. 2018;46:392. doi: 10.1016/j.jddst.2018.06.004

[26] Malikmammadov E, Tanir TE, Kiziltay A, et al. PCL and PCL-Based Materials in Biomedical Applications. J. Biomater. Sci. Polym. Ed. 2017;29(7–9):1–55. doi: 10.1080/09205063.2017.1394711 29053081

[27] Catalina M, Attenburrow GE, Cot J, et al. Influence of crosslinkers and crosslinking method on the properties of gelatin films extracted from leather solid waste. J Appl Polym Sci. 2010;119:2105. doi: 10.1002/app.32932

[28] Hulupi M, Haryadi H. Synthesis and Characterization of electrospinning PVA nanofiber-crosslinked by glutaraldehyde. Mater Today Proc. 2019;13:199. doi: 10.1016/j.matpr.2019.03.214

[29] Jayakrishnan A, Jameela SR. Glutaraldehyde as a fixative in bioprostheses and drug delivery matrices. Biomaterials. 1996;17(5):471. doi: 10.1016/0142-9612(96)82721-9 8991478

[30] Scheffel DLS, Soares DG, Basso FG, et al. Transdentinal cytotoxicity of glutaraldehyde on odontoblast-like cells. J Dent. 2015;43:997. doi: 10.1016/j.jdent.2015.05.004 25985981 PMC4509972

[31] Yang L, Yang Q, Lu DN. Effect of chemical crosslinking degree on mechanical properties of bacterial cellulose/poly(vinyl alcohol) composite membranes. Monatshefte Fur Chemie. 2014;145:91. doi: 10.1007/s00706-013-0968-9

[32] Anwar B, Bundjali B, Sunarya Y, et al. Properties of bacterial cellulose and its nanocrystalline obtained from pineapple peel waste juice. Fibers Polym. 2021;22:1228. doi: 10.1007/s12221-021-0765-8

[33] Nelson ML, O’Connor RT. Relation of certain infrared bands to cellulose crystallinity and crystal lattice type. Part II. A new infrared ratio for estimation of crystallinity in celluloses I and II. J Appl Polym Sci. 1964;8(3):1325. doi: 10.1002/app.1964.070080323

[34] Ditzel FI, Prestes E, Carvalho BM, et al. Nanocrystalline cellulose extracted from pine wood and corncob. Carbohydr Polym. 2017;157:1577. doi: 10.1016/j.carbpol.2016.11.036 27987871

[35] Ren -D, Yi H, Wang W, et al. The enzymatic degradation and swelling properties of chitosan matrices with different degrees of N-acetylation. Carbohydr. Res. 2005;340(15):2403–2410. doi: 10.1016/j.carres.2005.07.022 16109386

[36] Munawaroh HSH, Darojatun K, Gumilar GG, et al. Characterization of phycocyanin from Spirulina fusiformis and its thermal stability. J Phys Conf Ser. 2018;1013:012205. doi: 10.1088/1742-6596/1013/1/012205

[37] Liu R, Qin S, Li W. Phycocyanin: anti-inflammatory effect and mechanism. Biomed Pharmacother. 2022;153:113362. doi: 10.1016/j.biopha.2022.113362 36076518

[38] Munawaroh HSH, Gumilar GG, Nurjanah F. In-vitro molecular docking analysis of microalgae extracted phycocyanin as an anti-diabetic candidate. Biochem Eng J. 2020;161:107666. doi: 10.1016/j.bej.2020.107666

[39] Sala L, Figueira FS, Cerveira GP, et al. Kinetics and adsorption isotherm of C-phycocyanin from Spirulina platensis on ion-exchange resins. Brazilian J Chem Eng. 2014;31(4):1013. doi: 10.1590/0104-6632.20140314s00002443

[40] Batmaz R, (2013).

[41] Ayawei N, Ebelegi AN, Wankasi D. Modelling and interpretation of adsorption isotherms. J Chem. 2017;2017:1–11. doi: 10.1155/2017/3039817

[42] Kumar KV, Gadipelli S, Wood B, et al. Characterization of the adsorption site energies and heterogeneous surfaces of porous materials. J Mater Chem A. 2019;7:10104. doi: 10.1039/C9TA00287A

[43] Tsade Kara H, Anshebo ST, Sabir FK, et al. Removal of methylene blue dye from wastewater using periodiated modified nanocellulose. Int J Chem Eng. 2021;1:1–16. doi: 10.1155/2021/9965452

[44] Johar N, Ahmad I, Dufresne A. Extraction, preparation and characterization of cellulose fibres and nanocrystals from rice husk. Industrial Crops And Products. 2012;37:93. doi: 10.1016/j.indcrop.2011.12.016

[45] Kumar A, Singh Negi Y, Choudhary V, et al. Characterization of cellulose nanocrystals produced by acid-hydrolysis from sugarcane bagasse as agro-waste. J Mater Phys Chem. 2020;2(1):1–8. doi: 10.12691/jmpc-2-1-1

[46] Jeon JG, Kim HC, Palem RR, et al. Cross-linking of cellulose nanofiber films with glutaraldehyde for improved mechanical properties. Mater Lett. 2019;250:99. doi: 10.1016/j.matlet.2019.05.002

[47] Hou T, Guo K, Wang Z, et al. Glutaraldehyde and polyvinyl alcohol crosslinked cellulose membranes for efficient methyl orange and Congo red removal. Cellul. 2019;26(8):5065. doi: 10.1007/s10570-019-02433-w

[48] Liu L, Jiang T, Yao J. A two-step chemical process for the extraction of cellulose fiber and pectin from mulberry branch bark efficiently. J Polym Environ. 2011;19(3):568. doi: 10.1007/s10924-011-0300-x

[49] Wijaya CJ, Saputra SN, Soetaredjo, FE, et al. Cellulose nanocrystals from passion fruit peels waste as antibiotic drug carrier. Carbohydr Polym. 2017;175:370–376. doi: 10.1016/j.carbpol.2017.08.004 28917878

[50] Brinchi L, Cotana F, Fortunati E, et al. Production of nanocrystalline cellulose from lignocellulosic biomass: technology and applications. Carbohydr Polym. 2013;94:154. doi: 10.1016/j.carbpol.2013.01.033 23544524

[51] Oun AA, Rhim JW. Isolation of cellulose nanocrystals from grain straws and their use for the preparation of carboxymethyl cellulose-based nanocomposite films. Carbohydr Polym. 2016;150:187. doi: 10.1016/j.carbpol.2016.05.020 27312629

[52] Maiti S, Jayaramudu J, Das K, et al. Preparation and characterization of nano-cellulose with new shape from different precursor. Carbohydr Polym. 2013;98(1):562. doi: 10.1016/j.carbpol.2013.06.029 23987382

[53] Eriksen NT. Production of phycocyanin—a pigment with applications in biology, biotechnology, foods and medicine. Appl Microbiol Biotechnol. 2008;80(1):1–14. doi: 10.1007/s00253-008-1542-y 18563408

[54] Manna S, Roy D, Saha P, et al. Rapid methylene blue adsorption using modified lignocellulosic materials. Process Saf Environ Prot. 2017;107:346. doi: 10.1016/j.psep.2017.03.008

[55] R R, Thomas D, Philip E, et al. Potential of nanocellulose for wastewater treatment. Chemosphere. 2021;281:130738. doi: 10.1016/j.chemosphere.2021.130738 34004518

[56] Zhang Y, Zheng Y, Yang Y, et al. Mechanisms and adsorption capacities of hydrogen peroxide modified ball milled biochar for the removal of methylene blue from aqueous solutions. Bioresour Technol. 2021;337:125432. doi: 10.1016/j.biortech.2021.125432 34171704

[57] Saadi R, Saadi Z, Fazaeli R, et al. Monolayer and multilayer adsorption isotherm models for sorption from aqueous media. Korean J Chem Eng. 2015;32:787. doi: 10.1007/s11814-015-0053-7

[58] Hasan N, Rahman L, Kim SH, et al. Recent advances of nanocellulose in drug delivery systems. J Pharm Investig. 2020;50(6):553. doi: 10.1007/s40005-020-00499-4

[59] Qi W, Yu J, Zhang Z, et al. Effect of pH on the aggregation behavior of cellulose nanocrystals in aqueous medium. Mater Res Express. 2019;6:125078. doi: 10.1088/2053-1591/ab5974

[60] Kamida K, Kunihiko K, Matsui T, et al. Study on the solubility of cellulose in aqueous alkali solution by deuteration IR and 13C NMR. Polym J. 1984;16:857. doi: 10.1295/polymj.16.857

[61] Genta I, Costantini M, Asti A, et al. Influence of glutaraldehyde on drug release and mucoadhesive properties of chitosan microspheres. Carbohydr Polym. 1998;36:81. doi: 10.1016/S0144-8617(98)00022-8

[62] Jantarat C, Muenraya P, Srivaro S, et al. Comparison of drug release behavior of bacterial cellulose loaded with ibuprofen and propranolol hydrochloride. RSC Adv. 2021;11(59):37354. doi: 10.1039/D1RA07761A 35496416 PMC9043831

